# RD21-like proteases: key effector hubs in plant–pathogen interactions

**DOI:** 10.1093/jxb/erae496

**Published:** 2024-12-10

**Authors:** Jie Huang, Renier A L van der Hoorn

**Affiliations:** The Plant Chemetics Laboratory, Department of Biology, University of Oxford, Oxford OX1 3RB, UK; The Plant Chemetics Laboratory, Department of Biology, University of Oxford, Oxford OX1 3RB, UK; Swedish University of Agricultural Sciences, Sweden

**Keywords:** Inhibitor, papain-like cysteine proteases, plant immunity, plant protease, effector, RD21

## Abstract

Over the past decades, numerous studies have demonstrated that proteases serve as a crucial regulatory mechanism in controlling plant immunity. In this review, we specifically focus on the role of one subfamily of RD21-like papain-like cysteine proteases that carry a C-terminal granulin domain. These proteases share high homology but have been described under very different names in different plant species. We provide a comprehensive overview of the background, endogenous regulation, and subcellular localization of RD21-like proteases in plants. Notably, RD21-like proteases act in immunity against various pathogens and they are targeted by many unrelated pathogen-secreted effectors that inactivate, mislocalize, or degrade RD21-like proteases. We highlight open questions and strategies to use this knowledge to develop innovative approaches for crop protection.

## Teaser

RD21-like proteases act in immunity and are targeted by unrelated effectors produced by a diverse range of plant pathogens throughout the plant kingdom.

## Introduction

**Fig. 1.**
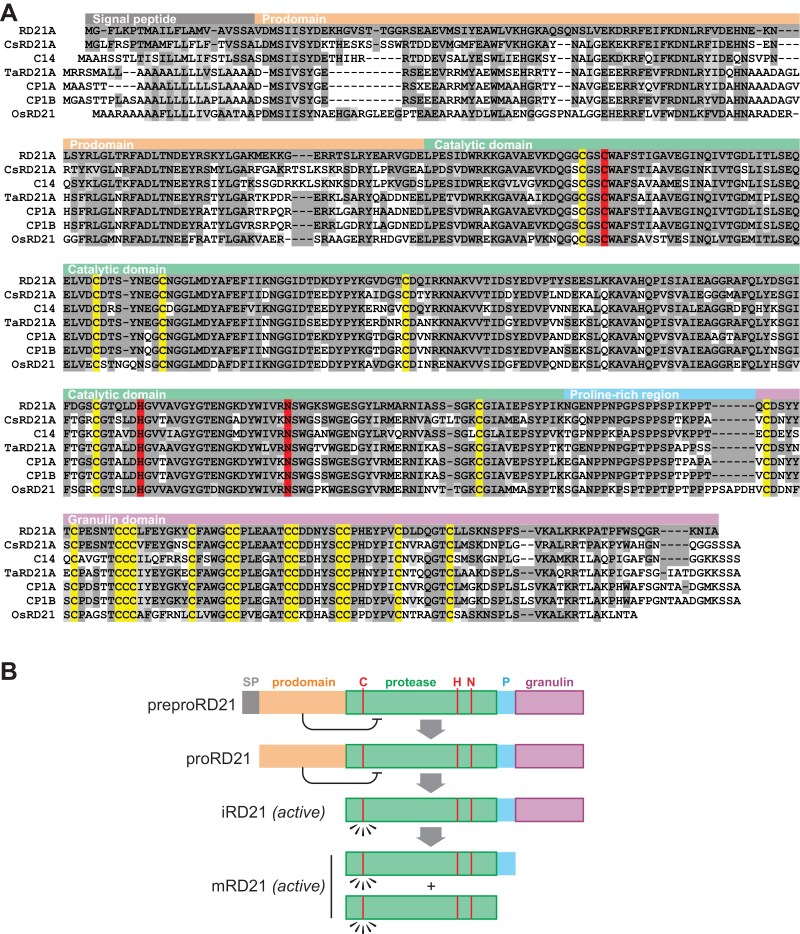
Protein sequence alignment of RD21 orthologues and maturation of RD21. (A) The sequence alignment includes *Arabidopsis thaliana* RD21; citrus *Cs*RD21A; tomato C14; wheat *Ta*RD21A; maize CP1A and CP1B; and rice *Os*RD21. Residues are highlighted if they are identical (dark grey), are similar (light grey) to those of RD21, or are catalytic residues (red) or Cys residues involved in disulfide bridges (yellow). (B) RD21 is encoded as a preproRD21 with a signal peptide (SP) that directs it into the secretory pathway, and a pro-domain that maintains the protease in an inactive state until its removal. RD21 is present in two active isoforms: the intermediate form (iRD21), which includes the granulin domain, and the mature form (mRD21), which comprises solely the protease domain, with or without the proline-rich (P) domain. The catalytic residues (C, H, N) are indicated with red lines.

Responsive-to-Desiccation-21 (RD21), encoded by the *RD21A* gene (At1g47128), was initially identified from a drought-responsive gene in Arabidopsis (Koizumi *et al.*, 1993): *RD21* is highly expressed in leaf tissue and is up-regulated during senescence (Koizumi *et al.*, 1993). Subsequent investigations have indicated that *RD21* is expressed across all organs of healthy plants and that transcript levels are up-regulated in response to both biotic and abiotic stress (van der Hoorn, 2013). RD21 is remarkably conserved throughout the plant kingdom, spanning monocots, dicots, and even gymnosperms (Fig. 1A). Orthologues of RD21 have been identified and characterized in tomato (known as C14, CYP1, TDI-65, or SENU3), rice (OsCP1, OCP, oryzain α and β), maize (CPPIC, Mir2, Mir3, CP1A, CP1B), wheat (Triticain α and β), and many other plants (van der Hoorn, 2013).

Papain-like cysteine proteases (PLCPs, MEROPS family C1A) in plants are classified into nine phylogenetic subfamilies (Richau *et al.*, 2012). PLCPs that carry a C-terminal granulin domain are only present in subfamily-1 (RD21-like proteases) and subfamily-4 (XBCP3-like proteases) (Richau *et al.*, 2012). RD21-like proteases consist of five distinct domains: a signal peptide, a 20 kDa autoinhibitory prodomain, a 33 kDa cysteine protease domain, a 2 kDa proline-rich domain, and a 10 kDa granulin domain (Gu *et al.*, 2012; van der Hoorn, 2013) (Fig. 1B). The N-terminal signal peptide targets RD21 to the endoplasmic reticulum lumen during translation. The prodomain carries an ERFNIN motif and acts as an inhibitor of the protease domain. The protease domain contains three catalytic residues (Cys, His, and Asn) and six additional conserved Cys residues that form three disulfide bridges: 1–3, 2–4 and 5–6. The fourth domain is rich in prolines. The granulin domain exhibits homology to granulin/epithelin proteins in animals, and is characterized by 14 conserved Cys residues that play a vital role in stabilizing its β-sheet hairpin fold structure (Bateman and Bennett, 2009). Several members of RD21-like proteases in subfamily-1 lack the C-terminal granulin domain (Richau *et al*., 2012), but these are not included in this review.

RD21 undergoes a multi-step maturation process involving three proteolytic events (Fig. 1B) (Yamada *et al.*, 2001). First, the signal peptide of preproRD21 is excised during translocation into the endoplasmic reticulum lumen, yielding proRD21. Subsequently, the prodomain of proRD21 is cleaved to produce intermediate RD21 (iRD21). This step is not autocatalytic and requires exogenous factors (Yamada *et al.*, 2001). Finally, the granulin domain is removed, leading to the formation of mature RD21 (mRD21; Gu *et al.*, 2012). Granulin domain removal requires the catalytic Cys and His residues and is therefore autocatalytic (Gu *et al.*, 2012). The fate of the granulin domain after cleavage as well as the cleavage site that removes the granulin domain remains elusive (Yamada *et al.*, 2001; Gu *et al.*, 2012). While both iRD21 and mRD21 exhibit proteolytic activity, iRD21 is thought to be less active due to its tendency to aggregate and precipitate at low PH (Yamada *et al.*, 2001).

## Four endogenous inhibitors are proposed to regulate RD21

Given that RD21 is a protease, precise regulation of its activity is important in plants (Rustgi *et al.*, 2017). The activity of RD21 is thought to be modulated by at least four endogenous protease inhibitors: a cystatin, a serpin, a Kunitz-type protease inhibitor, and protein disulfide isomerase. The remarkable activation of RD21-like proteases by SDS might be caused by the denaturation of endogenous inhibitors (Gu *et al.*, 2012) or its solubilization from aggregates.

There are several studies showing cystatins interacting with RD21. For example, in maize the RD21 orthologue (CPPIC) was co-purified with a cystatin, forming a protease-inhibitor complex in leaf extracts (Yamada *et al.*, 2000). In addition, a protein complex consisting of an RD21-like protease and an endogenous cystatin has been isolated from senescing spinach leaves (Tajima *et al.*, 2011). The second protein capable of inhibiting RD21 activity is serpin. Serpins are found in both animals and plants and feature a reactive centre loop (RCL) that displays a protease target sequence (Huntington, 2006). The cleavage of the RCL by Cys proteases leads to a conformational change in the serpin structure that dislocates the catalytic Cys from the catalytic triad, resulting in an irreversible, covalent bond between the serpin and the protease. The serpin–RD21 complex will form in extracts when cytoplasm-localized serpin is mixed with extracytoplasmic RD21 (Lampl *et al.*, 2010). Notably, both *rd21* and *atserpin1* knock-out mutants lacked the serpin-protease complex, suggesting that RD21 is a primary target of AtSerpin1 in leaf extracts (Lampl *et al.*, 2010). The third protein that inhibits RD21 activity is a Kunitz-type protease inhibitor that was initially identified as a water-soluble chlorophyll-binding protein (WSCP). Complexes between RD21 and WSCP accumulate in developing flowers and in the apical hook of plants undergoing skotomorphogenesis (Rustgi *et al.*, 2017). In contrast to the tight binding of RD21 to cystatin, or its irreversible binding to serpin, the interaction between RD21 and WSCP is reversible and relieved upon light exposure (Boex-Fontvieille *et al.*, 2015). Finally, a study on Arabidopsis protein disulfide isomerase-5 (PDI5) revealed its interaction with RD21 in yeast two-hybrid assays, and the interaction between PDI5 and RD21 was confirmed through co-immunoprecipitation. Moreover, electron microscopy studies indicated that PDI5 and RD21 co-localize in the endoplasmic reticulum, Golgi, and lytic vacuoles of cells during seed development (Ondzighi *et al.*, 2008). Although an *in vitro* cysteine protease assay suggested that PDI5 inhibits RD21 (Ondzighi *et al.*, 2008), it is notable that the only three PLCPs identified by yeast two-hybrid assays all contain a granulin domain, suggesting that PDI5 rather interacts with the Cys-rich granulin domain. Overall, these findings indicate that RD21 activity is tightly controlled after its pro-domain is removed.

## Subcellular localization of RD21

It is reported that Arabidopsis RD21 is localized in the vacuole, endoplasmic reticulum, endoplasmic reticulum bodies, Golgi, prevacuolar compartments and apoplast (Hayashi *et al.*, 2001; Yamada *et al.*, 2001; Carter *et al.*, 2004; Bozkurt *et al.*, 2011; Cui *et al.*, 2017). While RD21 is abundant in the vacuole (Yamada *et al.*, 2001; Carter *et al.*, 2004), there has been an ongoing debate regarding its transport route to reach the vacuole. The presence of RD21 in endoplasmic reticulum bodies indicates that RD21 comes directly from the endoplasmic reticulum (Hayashi *et al.*, 2001). However, the interaction between PDI5 and RD21, combined with their co-localization from the endoplasmic reticulum to the Golgi and eventually to the lytic vacuoles, provides compelling evidence that RD21 is transported through the Golgi to vacuoles (Ondzighi *et al.*, 2008). Similarly, by employing an introduced N-glycan sensor and conducting deglycosylation experiments, the vast majority of RD21 was found to pass through the Golgi upon transient expression (Gu *et al.*, 2012). However, although this observation suggests that RD21 traffics through the Golgi, this does not necessarily imply that RD21 localized in endoplasmic reticulum bodies follows the same route. Endoplasmic reticulum bodies are specific structures that occur in Arabidopsis cotyledons but are absent in adult leaves unless these are wounded (Hayashi *et al.*, 2001). Several reports also show that RD21-like proteases are secreted into the apoplast. C14/TDI-65, for instance, was detected as an active protease in the apoplast of tomato (Shabab *et al.*, 2008; Van Esse *et al.*, 2008). *Ta*RD21A is secreted into the apoplast, where it plays a crucial role in the antiviral response of wheat (Liu *et al.*, 2023). Recently, it was reported that *Os*RD21 is primarily localized in the plasma membrane (Liu *et al.*, 2024). Co-expression with RxLR effector AVRblb2 from *Phytophthora infestans* prevents the secretion of C14 and shifts its subcellular localization to the cell periphery, showing significant overlap with a plasma membrane marker (Bozkurt *et al.*, 2011). Tomato CYP1 and the V2 protein of tomato yellow leaf curl virus co-localize within the cytoplasm when co-expressed in tobacco protoplasts, indicating that V2 can mislocalize CYP1 (Bar-Ziv *et al.*, 2012).

## RD21s contribute to plant immunity

Multiple instances of protease depletion through RNA interference or knockout strategies indicate the importance of RD21 and its orthologues in plant immunity (Table 1). Arabidopsis *rd21* T-DNA insertion knock-out mutants are more susceptible to the fungal grey mould pathogen *Botrytis cinerea* when whole plants are infected (Shindo *et al.*, 2012). Likewise, *rd21* mutants are also more susceptible to the fungal anthracnose pathogen *Colletotrichum higgisianum* (Lampl *et al.*, 2013). Remarkably, the opposite reduced susceptibility phenotype with *B. cinerea* and the fungal white mould pathogen *Sclerotina sclerotiorum* was observed for the same *rd21* mutants in the detached leaf assays (Lampl *et al.*, 2013). Nevertheless, Arabidopsis *rd21* knock-out lines do not exhibit altered susceptibility to the oomycete downy mildew pathogen *Hyaloperonospora arabidopsidis* or the bacterial leaf spot pathogen *Pseudomonas syringae* (Shindo *et al.*, 2012). Moreover, Arabidopsis *rd21* mutants are more susceptible to the root-knot nematode *Meloidogyne chitwoodi* and *M. incognita* (Davies *et al.*, 2015; Yu *et al.*, 2024) and the protist clubroot pathogen *Plasmodiophora brassicae*, accompanied by a suppression of the defence response (Li *et al.*, 2024). Silencing the *NbC14* in *Nicotiana benthamiana* increases susceptibility to oomycete late blight pathogen *Phytophthora infestans* (Bozkurt *et al.*, 2011), but it later appeared that *Nb*C14 is not the orthologue of Arabidopsis RD21. Instead, *Nb*C14 belongs to the XBCP3-like protease subfamily-4 and was therefore renamed *Nb*CP14 (Paireder *et al.*, 2016), as it is an orthologue of tobacco *Nt*CP14, which plays a role in programmed cell death during embryo development (Zhao *et al.*, 2013). Interestingly, overexpression of *Os*RD21 in rice enhanced resistance to the fungal rice blast pathogen *Magnaporthe oryzae* but had no effect on infections with the fungal brown spot pathogen *Bipolaris oryzae* and bacterial leaf blight pathogen *Xanthomonas oryzae* (Liu *et al.*, 2024). Moreover, an oryzain α-chain precursor (OCP), the orthologue of RD21, is also involved in the regulation of resistance against three different *M. oryzae* isolates (97-27-2, JL021605, and ZB13), as evidenced by the shorter lesion length observed in *ocp* knockout lines (Li *et al.*, 2022). However, this *OCP* gene is not involved in the resistance to *X. oryzae* pv*. oryzae* (*Xoo*; Li *et al.*, 2022). Moreover, a recent study also showed that wheat *Ta*RD21A acts as a positive regulator of wheat resistance to wheat yellow mosaic virus infection (Liu *et al.*, 2023). In summary, RD21 proteins are essential for plant immunity against various biotic stresses in different plant species. Although these studies demonstrated the role of RD21-like proteases in immunity, the underlying mechanisms remain to be elucidated.

**Table 1.** RD21 and its orthologues involved in plant immunity

Name	Species	Phenotype and mechanism	References
RD21	Arabidopsis	*rd21* mutants (whole plants) are more susceptible to *Botrytis cinerea*	Shindo *et al.* (2012)
		No disease phenotypes were observed in *rd21* mutants infected with virulent or avirulent *Pseudomonas syringae* and *Hyaloperonospora arabidopsidis*	Shindo *et al.* (2012)
		*rd21* knockout mutants are more susceptible to *Colletotrichum higgisianum*	Lampl *et al.* (2013)
		*rd21* knockout (detached leaves) are more resistant to *Botrytis cinerea* and *Sclerotina sclerotiorum*	Lampl *et al.* (2013)
		*rd21* mutants are hypersusceptible to *Meloidogyne chitwoodi* and *Meloidogyne incognita*	Davies *et al.* (2015), Yu *et al.* (2024)
		*Meloidogyne chitwoodi* effector *Mc*1194 interacts with RD21	Davies *et al.* (2015)
		*Meloidogyne incognita* effector *Mi*CE108 physically associates with RD21, inhibits RD21 activity, and facilitates RD21 degradation	Yu *et al.* (2024)
		*Heterodera schachtii* effector *Hs*4E02 interacts with RD21, and targets RD21 to the nucleus and cytoplasm	Pogorelko *et al.* (2019)
		Mutant *rd21* lines are more susceptible to *Plasmodiophora brassicae*	Li *et al.* (2024)
		*Plasmodiophora brassicae* secreted E3 ubiquitin ligase PbE3-2 interacts with RD21 to trigger its proteasome-mediated degradation	Li *et al.* (2024)
C14	Potato	*Phytophthora infestans* effectors EpiC1 and EpiC2B interact with potato C14 and inhibit C14 activity.	Kaschani *et al.* (2010)
C14/CYP1	Tomato	EpiC1 and EpiC2B interact with C14 and inhibit C14 activity	Kaschani *et al.* (2010)
		Avrblb2 interacts with C14 and prevents C14 secretion	Bozkurt *et al.* (2011)
		*Pseudomonas syringae* effector Cip1 inhibits C14 activity	Shindo *et al.* (2016)
		TYLCV V2 protein interacts with C14/CYP1	Bar-Ziv *et al.* (2012)
		TYLCV V2 protein inhibits C14/CYP1 activity	Bar-Ziv *et al.* (2015)
*Os*RD21	Rice	*Os*RD21 overexpression enhances resistance to *Magnaporthe oryzae* but not to *Bipolaris oryzae* and *Xanthomonas oryzae*	Liu *et al.* (2024)
		*Magnaporthe oryzae* effector MoErs1 interacts with rice *Os*RD21 and inhibits its activity	Liu *et al.* (2024)
OCP	Rice	*ocp* knockout is more resistant to *Magnaporthe oryzae*, but not to *Xanthomonas oryzae*	Li *et al.* (2022)
*Ta*RD21A	Wheat	*Ta*RD21A knockout is more susceptible to WYMV	Liu *et al.* (2023)
		WYMV NIa interacts with TaRD21A and inhibits *Ta*RD21A activity	Liu *et al.* (2023)
CP1A/CP1B	Maize	*Ustilago maydis* effector protein Pit2 interacts with and inhibits CP1A and CP1B	Mueller *et al.* (2013)
		Processing of Pit2 releases embedded inhibitor peptide	Misas Villamil *et al.* (2019)
*Cs*RD21A	Citrus	SDE1 from ‘*Candidatus* Liberibacter asiaticus’ interacts with *Cs*RD21A and inhibits its activity	Clark *et al.* (2018)

TYLCV, tomato yellow leaf curl virus; WYMV, wheat yellow mosaic virus.

## RD21s are common targets of various pathogens

**Fig. 2.**
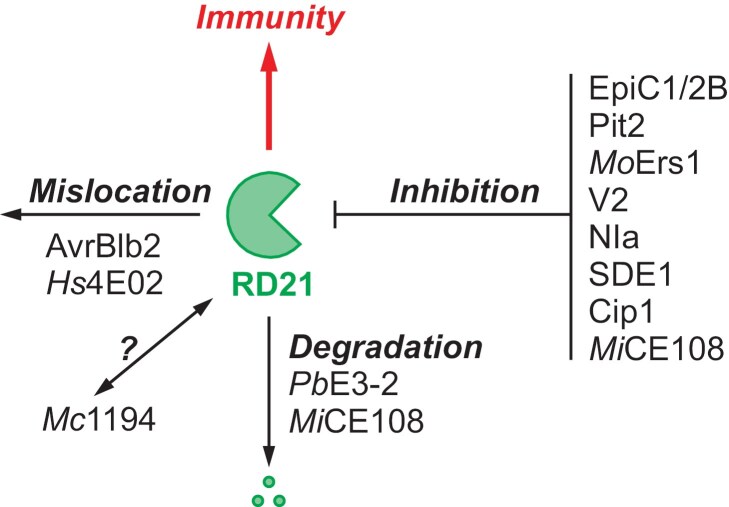
Pathogen-secreted effectors manipulate RD21-like proteases in different ways. Effectors that interact with RD21 and its orthologues can inhibit RD21, altering its subcellular localization, or triggering its degradation. The mode of action of *Mc*1194 on RD21 is unknown.

Various plant microbes, such as fungi, bacteria, oomycetes, nematodes, and viruses, exploit a diverse array of effectors to target RD21 and its orthologous proteins across different plant species, including Arabidopsis, tomato, maize, citrus, rice, and wheat (Fig. 2). Arabidopsis RD21, for instance, is targeted by four unrelated effectors. The root-knot nematode *Meloidogyne chitwoodi* secretes effector protein *Mc*1194 to interact with both the protease and granulin domain of Arabidopsis RD21 (Davies *et al.*, 2015). Likewise, sugar beet cyst nematode *Heterodera schachtii* effector *Hs*4E02 interacts with RD21 (Pogorelko *et al.*, 2019). Moreover, the root-knot nematode *Meloidogyne incognita* effector *Mi*CE108 physically associates with RD21 (Yu *et al.*, 2024). E3 ubiquitin ligase *Pb*E3-2 secreted by *P. brassicae* also interacts with RD21 (Li *et al.*, 2024). Tomato C14 (also called CYP1) is also targeted by various pathogen effectors. For instance, tomato and potato C14 interact with cystatin-like effectors EpiC1 and EpiC2B secreted by *P. infestans* (Kaschani *et al.*, 2010) and this pathogen also secretes RxLR effector AvrBlb2 that associates with tomato C14 (Bozkurt *et al.*, 2011). Similarly, tomato C14 is targeted by the RNA-silencing suppressor V2 of the tomato yellow leaf curl geminivirus (Bar-Ziv *et al.*, 2012). In addition, RD21 orthologues from maize, citrus, rice, and wheat are also targeted by various unrelated effectors. For example, effector protein Pit2 secreted by the fungal maize smut pathogen *Ustilago maydis* interacts with maize RD21-like proteases CP1A and CP1B (Mueller *et al.*, 2013). The inhibitory function of Pit2 depends on a conserved motif consisting of 14 amino acids that is activated upon cleavage (Misas Villamil *et al.*, 2019). Sec-delivered effector-1 (SDE1) from the Huanglongbing bacterium *Candidatus* Liberibacter asiaticus (CLas) directly interacts with citrus *Cs*RD21a (Clark *et al.*, 2018), and cytoplasmic effector *Mo*Ers1 of *M. oryzae* interacts with rice *Os*RD21 (Liu *et al.*, 2024). In addition, nuclear inclusion protease-a (NIa) of wheat yellow mosaic virus interacts with wheat *Ta*RD21A (Liu *et al.*, 2023). In summary, RD21 and its orthologues are common targets for diverse microbe-secreted effectors in many plant species.

**Fig. 3.**
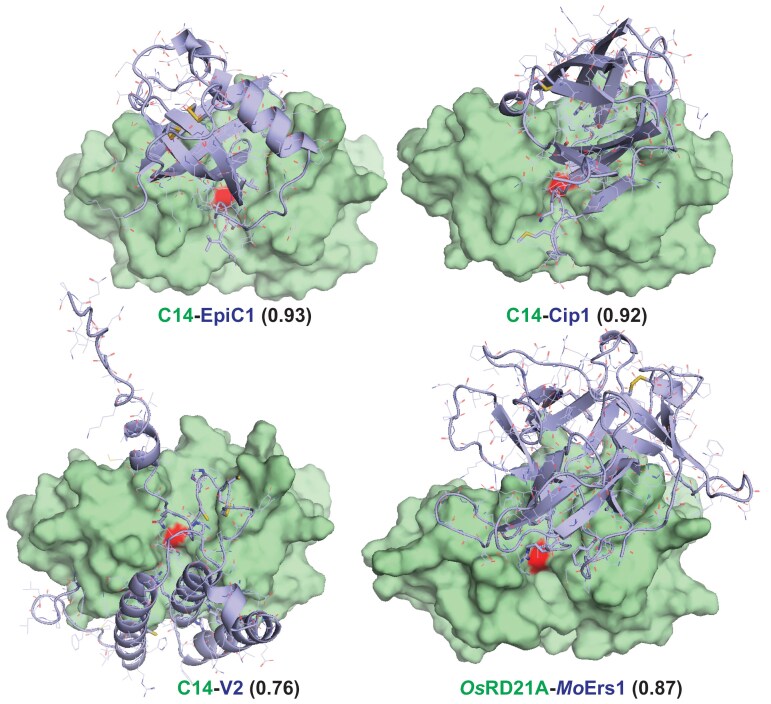
AlphaFold Multimer (AFM)-predicted models of RD21-like proteases and four different inhibitors. RD21 and its orthologues are shown in a pale green surface representation with the active site (red) and inhibitors are shown in light blue as cartoon and lines with disulfides and interface residues shown as sticks. AFM scores and PDB files of these models are available in Supplementary Table S1 and Supplementary Dataset S1, respectively.

The diversity of RD21-targeting inhibitors is also illustrated with structural predictions. Structural models generated with AlphaFold Multimer (AFM) indicated that four inhibitors block the active site of RD21 and its orthologues in different ways (Fig. 3). Complexes of the other protein pairs received relatively low confidence scores (Supplementary Table S1), despite their reported interactions, indicating that AFM may produce false negatives (Homma *et al.*, 2024).

## Effectors use different strategies to interfere in RD21 function

The interactions of effectors with RD21 and its orthologues can result in its inhibition, mislocalization, and degradation (Fig. 2). Most described effectors are protease inhibitors. For instance, RD21 and its orthologues are inhibited by *Meloidogyne incognita* effector *Mi*CE108 (Yu *et al.*, 2024), *P. infestans* cystatin-like effectors EpiC1 and EpiC2B (Kaschani *et al.*, 2010), *Pseudomonas syringae* pv. *tomato* DC3000 chagasin-like effector Cip1 (Shindo *et al.*, 2016), *Ustilago maydis* effector protein Pit2 (Mueller *et al.*, 2013; Misas Villamil *et al.*, 2019), *Candidatus* Liberibacter asiaticus effector SDE1 (Clark *et al.*, 2018), *Magnaporthe oryzae* effector *Mo*Ers1 (Liu *et al.*, 2024), wheat yellow mosaic virus NIa protein and tomato yellow leaf curl virus V2 protein (Bar-Ziv *et al.*, 2015; Liu *et al.*, 2023). By contrast, two effectors alter the subcellular localization of RD21 and its orthologues. *Phytophthora infestans* RxLR effector AvrBlb2 prevents secretion of the C14 into the apoplast, accumulating these vesicles around haustoria instead (Bozkurt *et al.*, 2011). By contrast, the *Heterodera schachtii* effector *Hs*4E02 targets RD21 to the nucleus and cytoplasm instead of the vacuole (Pogorelko *et al.*, 2019). In addition, two effectors trigger RD21 degradation. *Meloidogyne incognita* effector *Mi*CE108 facilitates the protein degradation of Arabidopsis RD21 via the endosomal-dependent pathway or the proteasome (Yu *et al.*, 2024). Likewise, *Plasmodiophora brassicae* effector *Pb*E3-2 is a RING-type E3 ubiquitin ligase that directly ubiquitinates Arabidopsis RD21, leading to its subsequent degradation through the proteasome (Li *et al.*, 2024). Overall, these interactions demonstrate the sophisticated strategies employed by plant pathogens to manipulate the function of RD21-like proteases to subvert plant immune responses.

## Open questions and prospects

In this review, we emphasized the importance of RD21 and its orthologues in plant immunity. However, numerous open questions about RD21s remain. We outline here some interesting questions we anticipate resolving in the future, along with additional intriguing topics for further investigation.

(i) How does RD21 act in immunity? Do RD21-like proteases harm pathogens directly by degrading their proteins, or are they involved in cellular processes that provide immunity, such as endogenous protein trafficking? The broad proteolytic activity supports the first hypothesis, but its mostly intracellular location supports the latter hypothesis.
(ii) Why does RD21 have such an important role in immunity? PLCPs in plants are classified into nine phylogenetic subfamilies: why do RD21-like proteases have such a central role in immunity over other PLCPs? Why do all these effectors from evolutionarily unrelated pathogens target RD21-like proteases? It would be interesting to answer these questions in the future.
(iii) What are the substrates of RD21-like proteases? Despite numerous studies highlighting the role of RD21-like proteases in immunity, biologically relevant substrates remain to be elucidated. Innovative technologies like high-efficiency undecanal-based N termini enrichment (HUNTER) (Weng *et al.*, 2019) and protease trap assays (Tang *et al.*, 2022) will undoubtedly be helpful to identify substrates from both plant and pathogen and reveal underlying molecular mechanism of RD21-mediated immunity.
(iv) Do all pathogens target RD21-like proteases? We summarized 13 effectors that target RD21 and its orthologues in different ways and these effectors are produced by viruses, bacteria, protists, oomycetes, fungi, and nematodes. It seems likely that many (if not most) pathogens will employ effectors that target RD21-like proteases, and uncover novel mechanisms.
(v) What is the role of the granulin domain of RD21? The granulin domain facilitates the insolubility of the iRD21 isoform and is autocatalytically removed to release soluble mRD21. The fate of the released granulin domain is unknown, but it is thought to be stable given its compact fold, stabilized by disulfide bridges. The granulin domain also exists in animals in tandem repeats but its C-terminal fusion to a protease is unique to the plant kingdom. Animal granulins are associated with protein homeostasis, development, and inflammation but the underlying molecular mechanisms are unresolved (Bateman and Bennett 2009).
(vi) Does RD21-like protease undergo phase separation? The granulated, intermediate RD21 (iRD21) precipitates at low pH and presumably resides in aggregates in the vacuole and apoplast (Yamada *et al.*, 2001). These biomolecular condensates could have significant implications for the function of RD21 in immunity. Identification of other components in these iRD21 condensates and elucidating the distinct roles of iRD21 in these aggregates and mRD21 outside these aggregates are interesting topics for further studies.
(vii) Is there a role for transpeptidase activity? Previous research has demonstrated that RD21 is capable of transpeptidation, using β-lactone probes and peptides as donor molecules, which results in the N-terminal transpeptidation of Arabidopsis proteins at neutral/basic pH (Wang *et al.*, 2008). Similar transpeptidation reactions were recently discovered in extracts of *Chlamydomonas reinhardtii*, catalysed by RD21-like protease *Cr*CEP1 (van Midden *et al.*, 2024). It will be interesting to identify the transpeptidation products produced *in vivo* and to investigate possible functions of these novel proteins.
(viii) How are RD21-like proteases regulated endogenously? The broad proteolytic activity of RD21-like proteases calls for a tight control over RD21 activity. Endogenous inhibitors, the presence of the C-terminal granulin domain, and the subcellular location and microenvironment (e.g. pH, redox) will all contribute to the regulation of RD21 activity, but their relative importance remains to be investigated.
(ix) Can we avoid RD21 manipulation to improve crop resistance? Various approaches can be taken to avoid RD21 manipulation and improve crop resistance. FY21001, for instance, is a designer compound that binds to and inactivates MoErs1, thereby disrupting the MoErs1–*Os*RD21 interaction and controlling rice blast (Liu *et al.*, 2024). In addition, natural variants of *Ta*RD21A confer resistance to wheat yellow mosaic virus infection in wheat (Liu *et al.*, 2023), indicating that the engineering of RD21 proteases is a viable strategy to increase crop resistance. This strategy is similar to the engineering of an EpiC2B-insensitive immune protease Pip1 that enhances resistance to *P. infestans* (Schuster *et al.*, 2024).

## Supplementary data

The following supplementary data are available at *JXB* online.

Table S1. AlphaFold Multimer scores of reported RD21-inhibitor interactions.

Dataset S1. PyMol.pse files of the four protease-inhibitor models shown in Fig. 3.

erae496_suppl_Supplementray_Table_S1

erae496_suppl_Supplementray_Dataset_S1
