# Supplementary material for: RD21-like proteases: key effector hubs in plant–pathogen interactions
Source: J Exp Bot. 2024 Dec 10;76(15):4212–9. doi: 10.1093/jxb/erae496 (PMC12485362; doi:10.1093/jxb/erae496)
Supplement: erae496_suppl_Supplementray_Table_S1 [file erae496_suppl_supplementray_table_s1.pdf]

Supplementary Table S1 AFM scores of reported RD21-inhibitor interactions

	**Model-1**	**Model-2**	**Model-3**	**Model-4**	**Model-5**
*At*RD21A_Hs4E02	0.335159909	0.384624186	0.323879607	0.352692475	0.386425233
*At*RD21A_MiCE108	0.536292048	0.671857744	0.273372229	0.476285705	0.417382831
*At*RD21A_PbE3-2	0.216649916	0.201596716	0.203201109	0.204546638	0.205308776
C14_Cip1	**0.917108663**	**0.908559142**	**0.892996793**	**0.896215347**	**0.900296875**
C14_EPIC1	**0.934153017**	**0.934156685**	**0.926201446**	**0.930990955**	**0.932494089**
C14_EPIC2B	**0.932636858**	**0.929880653**	**0.923249073**	**0.922811391**	**0.931365855**
C14_V2	0.325895059	0.270668125	0.28491674	0.27827978	**0.756164606**
CP1A_Pit2	0.311412775	0.303487047	0.300893106	0.314114741	0.293411517
CP1B_Pit2	0.31762299	0.310927944	0.316001003	0.310315994	0.296712421
*Cs*RD21A_SDE1	0.445046508	0.47315632	0.469558895	0.402924149	0.407535503
*Os*RD21A_MoErs1	0.465936813	0.27931419	0.654131256	**0.868177063**	**0.823071329**
*Ta*RD21A_NIa	0.381617995	0.406387567	0.422475569	0.410573523	0.322501746

The top five AlphaFold-Multimer predictions are generated as previously described (Homma et al., 2023). Shown are [0.8 ipTM + 0.2 pTM] scores of the top 5 models. Scores >0.75 are highlighted in bold. The complex between *At*RD21A and Mc1194 could not be predicted by AlphaFold-Multimer and is therefore not displayed here.

Homma, F., Huang, J. and van der Hoorn, R.A.L. (2023) AlphaFold-Multimer predicts cross-kingdom interactions at the plant-pathogen interface. *Nat Commun.* 14, 6040.
